# RNF173 suppresses RAF/MEK/ERK signaling to regulate invasion and metastasis via GRB2 ubiquitination in Hepatocellular Carcinoma

**DOI:** 10.1186/s12964-023-01241-x

**Published:** 2023-08-25

**Authors:** Jie Zhou, Daoyuan Tu, Rui Peng, Yuhong Tang, Qiangwei Deng, Bingbing Su, Shunyi Wang, Hao Tang, Shengjie Jin, Guoqing Jiang, Qian Wang, Xin Jin, Chi Zhang, Jun Cao, Dousheng Bai

**Affiliations:** 1 Department of Hepatobiliary Surgery, Clinical Medical College, Yangzhou University, Yangzhou, 225009 China; 2 Subei People’s Hospital Hepatobiliary Surgery. Institute of General Surgery, Yangzhou, 225001 China; 3 Biobank, Clinical Medical College, Yangzhou University, Northern Jiangsu People’s Hospital, Yangzhou, China

**Keywords:** Hepatocellular carcinoma, RNF173, GRB2, Ubiquitination, RAF/MEK/ERK signaling pathway

## Abstract

**Background:**

The role of the membrane-associated RING-CH (MARCH) family in carcinogenesis has been widely studied, but the member of this family, RNF173, has not yet been thoroughly explored in the context of hepatocellular carcinoma (HCC).

**Methods:**

With the use of an HCC tissue microarray and IHC staining, we aim to determine the differential expression of RNF173 in HCC patients and its clinical significance. The biological role of RNF173 is investigated through in vitro and in vivo experiments. RNA sequencing, mass spectrometry, and immunoprecipitation are performed to uncover the underlying mechanism of RNF173's impact on the development of HCC.

**Results:**

The mRNA and protein levels of RNF173 were significantly lower in HCC tissues than in normal tissues. HCC patients with low RNF173 expression had shorter overall survival and recurrence-free survival, and RNF173 was significantly correlated with tumor number, tumor capsule, tumor differentiation, and BCLC stage. In addition, in vitro and in vivo experiments showed that RNF173 downregulation exacerbated tumor progression, including migration, invasion, and proliferation. GRB2 is a key molecule in the RAF/MEK/ERK pathway. RNF173 inhibits the RAF/MEK/ERK signaling by ubiquitinating and degrading GRB2, thereby suppressing HCC cell proliferation, invasion and migration. Combining clinical samples, we found that HCC patients with high RNF173 and low GRB2 expression had the best prognosis.

**Conclusion:**

RNF173 inhibits the invasion and metastasis of HCC by ubiquitinating and degrading GRB2, thereby suppressing the RAF/MEK/ERK signaling pathway. RNF173 is an independent risk factor for the survival and recurrence of HCC patients. RNF173 may serve as a novel prognostic molecule and potential therapeutic target for HCC.

Video Abstract

**Graphical Abstract:**

Graphical abstract Model of RNF173 on RAF/MEK/ERK signaling. RNF173 knockdown resulted in impaired ubiquitination and degradation of GRB2, leading to the activation of the RAF/MEK/ERK signaling pathway and promotion of invasion and metastasis in HCC cells.

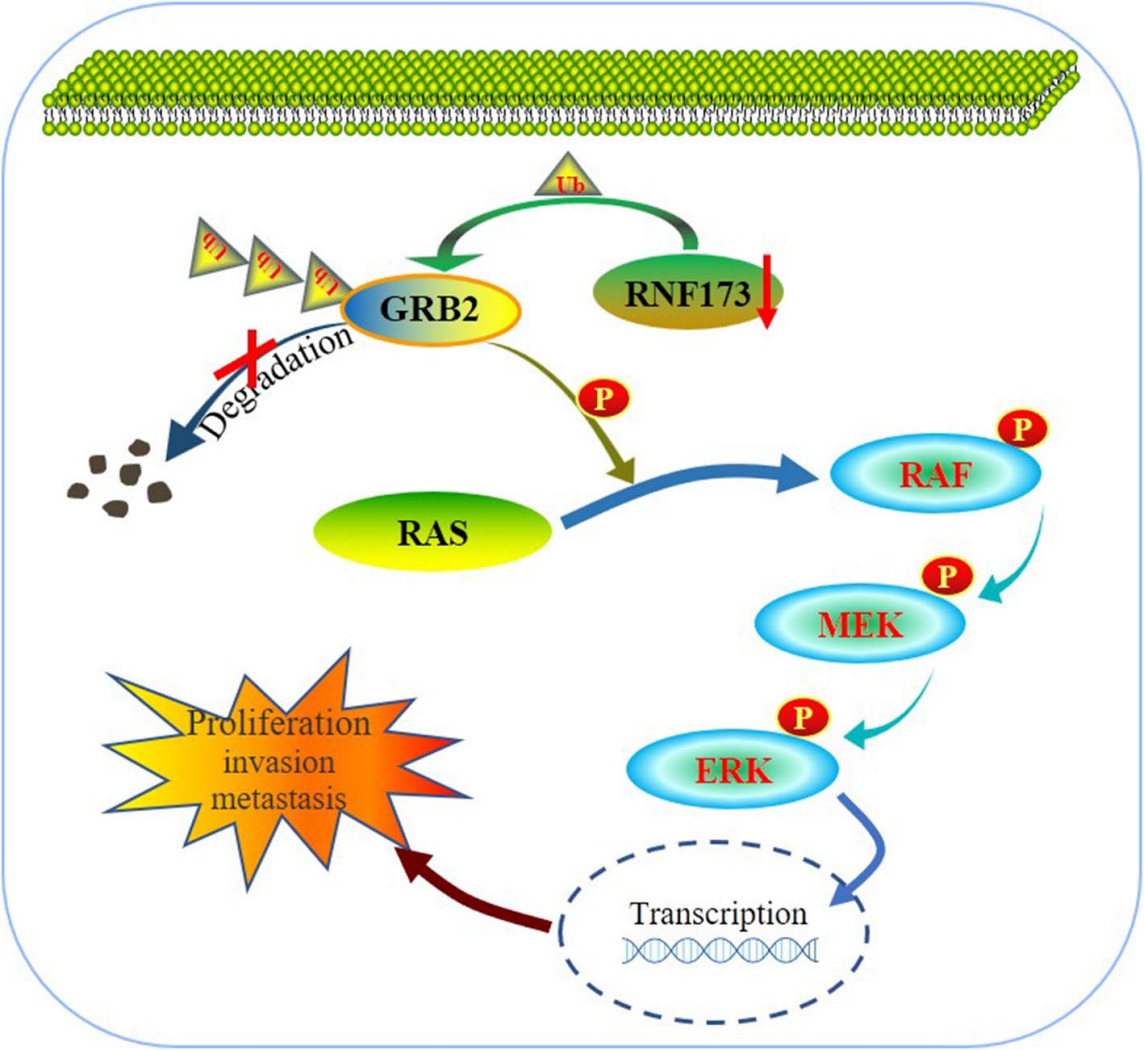

**Supplementary Information:**

The online version contains supplementary material available at 10.1186/s12964-023-01241-x

## Background

Hepatocellular carcinoma (HCC) is the third leading cause of cancer-related deaths globally, with five-year survival rate less than 20% [1]. The main risk factors for HCC are hepatitis virus and alcohol abuse [2]. Despite surgical resection being an option for early-stage HCC patients, most treatment strategies are limited for those diagnosed at an advanced stage [3]. Recent years have seen promising developments in the treatment of HCC patients with targeted drugs and immune checkpoint blockade (ICB) [4, 5]. However, despite these advancements, the majority of patients do not derive maximum benefit from systemic therapeutic agents [6, 7]. Therefore, it is imperative to identify the molecular mechanisms that underlie the occurrence and progression of HCC. In particular, effective biomarkers need to be discovered that can aid in improving the prognosis of HCC patients.

Ubiquitination, a crucial cellular process involved in regulating protein levels, has been implicated in various cellular functions such as cell metabolism, DNA damage, and the cell cycle [8]. E3 ubiquitin ligases play a critical role in this process by transferring ubiquitin tags to substrate proteins through E2-ubiquitin-binding enzymes, thereby controlling the quantity and quality of these proteins [9]. E3 dysregulation has been observed in tumor tissues, leading to the abnormal activation of carcinogenic or tumor suppressor pathways [10, 11]. The MARCH ligases, a subgroup of E3 ubiquitin ligases, have been shown to be involved in immune response and transmembrane trafficking [12]. Our recent research has suggested that MARCH family protein, including RNF173 (MARCH3), may have therapeutic significance for HCC patients undergoing chemotherapy, ICB, and TACE [13]. RNF173 has been found to have a potential role in restraining IL-6-induced STAT3 activation and inflammation-associated carcinogenesis in colorectal carcinoma [14]. RNF173 also negatively regulates the IL-1β inflammatory signal by catalyzing lysosomal-related degradation through K48-linked polyubiquitination of the IL-1 receptor [15]. Furthermore, RNF173 promotes the polyubiquitination and degradation of IL-5 and IL-3 receptor components following inflammation stimulation [16, 17]. RNF173 may play a crucial role in the malignant transformation of tumor cells in HCC. However, the role and mechanism of RNF173 in liver cancer remain to be studied.

Our study aimed to examine the expression of RNF173 in HCC tissues and its impact on patient prognosis, with a focus on understanding the precise mechanism by which RNF173 influences tumor progression.

## Methods

### Clinical specimens and ethics statement

140 cases of pathological tissues from liver cancer patients who underwent surgical resection at Subei People's Hospital of Jiangsu Province from January 2015 to December 2021 were collected, excluding patients who received TACE or systemic treatment before surgery. Shanghai Xinchao Biotechnology Co., Ltd. was commissioned to make tissue microarrays, which included both tumor tissues and matched adjacent non-tumor tissues. Tumor staging was performed according to the internationally recognized Barcelona Clinic Liver Cancer (BCLC) staging system. The use of the above HCC samples was approved by the Ethics Committee of Subei People's Hospital of Jiangsu Province with patient informed consent.

### Immunohistochemistry (IHC)

The HCC tissue microarrays were first placed in a 60 °C oven for 3 h, followed by immersion in xylene twice for 15 min each to remove paraffin. The tissues were then sequentially dehydrated in 100%, 95%, 85%, and 70% ethanol for 5 min each. The tissues were circled with a PAP pen, followed by adding an appropriate amount of 3% hydrogen peroxide solution for 10 min. Next, the tissues were circled with a PAP pen and incubated with 3% hydrogen peroxide solution for 10 min. Tissues were then subjected to antigen retrieval by immersion in preheated Tis-EDTA solution at 95 °C for 15 min, and allowed to cool to room temperature. Super-blocking solution (Absin) was used to block the tissues for 7 min, followed by incubation with a pre-diluted primary antibody at 4 °C overnight. An appropriate amount of enzyme-labeled secondary antibody was added on the tissues for 30 min. Enzyme-labeled secondary antibody was added to the tissues for 30 min, and signal development was achieved using diphenylamine, followed by counterstaining with hematoxylin. Tissues were dehydrated in the ethanol with elevated gradient and sealed with coverslips. The tissues were then dehydrated in increasing concentrations of ethanol and sealed with coverslips. The IHC methodology and qualitative staining intensity criteria were consistent with those previously reported in our study [18]. The ImageJ software was employed to calculate and analyze the AOD (average optical density) values for further analysis.

### Western blotting (WB) and real time quantitative PCR (qRT-PCR) analysis

For Western blotting analysis, tumor samples or cells were lysed in a buffer containing 1 mM PMSF and 1 mM phosphatase inhibitor (Beyotime). The lysates were centrifuged at 12000 g for 10 min, and protein concentrations were determined using the BCA method. Samples were boiled with SDS-PAGE sample loading buffer (Beyotime), loaded onto an SDS/PAGE gel, and transferred onto a PVDF membrane. After pre-treating the membrane with methanol, it was blocked with 5% BSA (Bovine Serum Albumin, Beyotime) for 1 h. The membrane was then incubated with the primary antibody at 4 °C overnight, followed by incubation with the corresponding secondary antibody for 1 h. The ECL was added, and the results were visualized using the ChemiDoc XRS + imager. Quantitative analysis was performed using Image J. The supplementary materials provide information for the primary and secondary antibodies. For RNA extraction from tissue or cell lines and subsequent quantitative real-time PCR (qRT-PCR), we followed the procedure described in our previous study [19]. The PCR primers were detailed in the supplementary materials.

### Immunofluorescent staining

Cells were cultured on sterile glass coverslips for 48 h, then fixed with 4% paraformaldehyde for 10 min and permeabilized with 0.1% Triton X-100 for 5 min. After blocking for 1 h and incubating with primary and secondary antibodies for 1 h each, the cells were washed and stained with Antifade Mounting Medium with DAPI (Beyotime). Confocal images were captured using a fluorescence orthomicroscope.

### Cell lines and transfection

The liver cancer cell lines HepG2, SK-Hep1, Huh7, and LM3 were obtained from the cell bank of the Chinese Academy of Sciences (Shanghai, China). The complete culture medium was composed of DMEM medium (HyClone) supplemented with 10% fetal bovine serum (FBS, Gibco) and 1% streptomycin/penicillin (Beyotime). The cell lines were maintained in a 37 °C, 5% CO2 incubator. The vectors used in this study were purchased from Shanghai Genomeditech Company (Shanghai, China). The target sequences for the RNF173 shRNA were listed in the supplementary materials. The stably transfected cells were selected using puromycin. The RAF inhibitor Y3009120 and the MEK inhibitor PD98059 were diluted in dimethyl sulfoxide (DMSO) for use in the experiments. The inhibitor concentration used in the cell experiments was 10 μM and the cell culture time was 6 h.

### Colony formation, Cell migration, invasion, and wound healing

Stably transfected cell lines with RNF173 shRNA or those overexpressing RNF173 and their respective control groups were cultivated in six-well plates for a period of 14 days. The cells were then fixed to the plates using paraformaldehyde for 30 min and subsequently stained with 0.1% crystal violet for an additional 30 min. Colony formation was evaluated by photography and quantification following air-drying at room temperature. The cell migration and invasion assays were conducted utilizing Transwell chambers in 24-well plates, with or without matrigel. A total of 1 × 10^5^ cells were seeded into the upper chamber in the absence of serum, with 700μL of complete medium present in the lower chamber. Fixation and staining procedures were consistent with those used in the colony formation assay. The wound healing assay involved the use of six-well plates, where a scratch was created using a 10μL pipette tip. Photographic documentation of the wound healing process was obtained at 0 h and 36 h.

### Animal model

The four-week-old nude mice used in this study were provided by the Translational Medical Center at Yangzhou University. HepG2 cells were digested using trypsin to prepare a cell suspension, and approximately 1 × 10^6 cells were subcutaneously injected into the mice's back. When the tumor grew to a diameter of 0.5 cm, the mice were anesthetized with isoflurane, and the tumor was removed and divided into 0.1 cm*0.1 cm pieces. These tumor tissues were then transplanted into the livers of new mice to establish an orthotopic tumor model. An in vivo imaging system was used to capture fluorescent images of tumors. Tumor samples were fixed in formalin for subsequent IHC experiments. The experiment was conducted in compliance with the guidelines established by the Animal Ethics Committee of the Clinical Medical College of Yangzhou University.

### Coimmunoprecipitation (co-IP) and mass spectrometry

The cells were first cleaned in PBS and lysis buffer containing 1 mM PMSF was added. The mixture was then stored at 4 °C for 15 min. The resulting lysate was collected into 1.5 mL tubes and subjected to ultrasonication for 5 s followed by centrifugation at 14,000 g for 10 min at cryogenic temperatures. The supernatant was then mixed with 5 μg of primary antibody (FLAG; GRB2 or IgG) and incubated overnight, followed by addition to protein A and protein agarose beads for a period of 3 h. After three wash cycles, the lysate was boiled in 1 × SDS loading buffer for 5 min. The resulting protein sample was utilized for subsequent Western blot analysis. The obtained immune coprecipitates commissioned Shanghai Genechem Company for mass spectrometry analysis.

### RNA-sequencing

Total RNA was extracted from stably transfected cells expressing shRNA-encoding lentiviruses (*n* = 3 independent samples per condition) and the corresponding negative control group. The samples were then sent to Majorbio Co., Ltd. for RNA-sequencing analysis. The mRNA was initially randomly fragmented using a fragmentation buffer. The library was constructed and subjected to Illumina sequencing for image acquisition and base calling. The resulting data was mapped to the reference genome, allowing for the examination of differential gene expression.

### Ubiquitination and cycloheximide (CHX) chase assay

The ubiquitination assay was performed on SK-Hep1-NC and SK-Hep1-shRNA3 cells treated with 5 μmol of MG132 (Selleck, China). Protein extracts from these cells were subjected to co-immunoprecipitation and Western blot analysis to detect ubiquitin. To determine the half-life of GRB2, a CHX chase assay was conducted. SK-Hep1-NC and SK-Hep1-shRNA3 cells were treated with CHX (50 μg/mL) for the appropriate duration, and Western blot analysis was performed as previously described.

### Statistical analysis

Statistical analysis was performed using SPSS 26.0 and Graphpad Prism 8. The Student's t-test was utilized to compare two groups of quantitative data, while the Pearson correlation coefficient was employed to evaluate correlation. Kaplan–Meier analysis was used to calculate overall survival and recurrence rate, and a Cox regression analysis was conducted to determine the independent prognostic factors. Significance was established at a *p*-value < 0.05.

## Results

### RNF173 is downregulated in HCC tissues and associated with poor prognosis in HCC patients

**Fig. 1:**
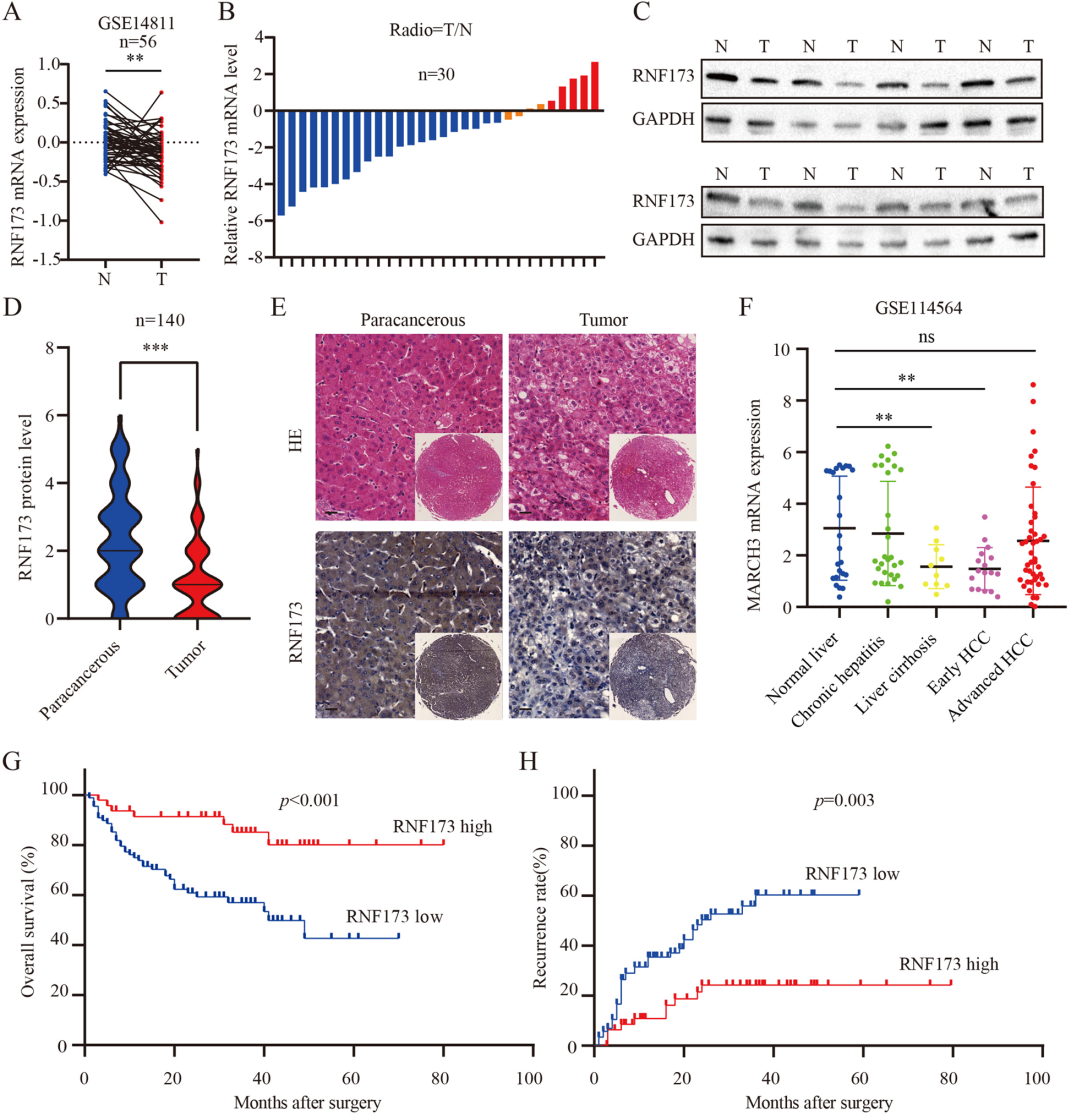
Downregulation of RNF173 is Associated with Poor Prognosis in HCC Patients. **A** RNF173 mRNA levels are significantly higher in adjacent tissues compared to tumor tissues in the GSE14811 dataset. **B** The mRNA expression of RNF173 in normal, chronic hepatitis, cirrhosis, early HCC, and advanced HCC in the GSE114564 dataset. **C** qRT-PCR validation of RNF173 mRNA expression, presented as log(T/N). **D** Western blot analysis of RNF173 protein expression in HCC tumor and matched adjacent tissues. **E** Immunohistochemical analysis and scoring of RNF173 expression in 140 HCC patients using tissue microarray. **F** Representative images of RNF173 expression by immunohistochemical staining. **G**, **H** Kaplan–Meier survival analysis of OS and RFS in HCC patients stratified by RNF173 expression levels. Scale bar: 100 μm. **p < 0.01, ***p < 0.001

The aim of this study was to investigate the role of RNF173 in hepatocellular carcinoma (HCC). We first analyzed the mRNA levels of RNF173 in HCC and adjacent tissues using the dataset GSE148111 from the GEO database and found that the mRNA level of RNF173 was significantly higher in adjacent tissues than in tumor tissues (Fig. 1C, *p* < 0.01). In addition, we further analyzed the expression of RNF173 in normal liver, chronic hepatitis, liver cirrhosis, early-stage HCC, and advanced-stage HCC in GSE114564 (Fig. 1B). The results showed that the expression of RNF173 gradually decreased from normal liver to early HCC but unexpectedly increased in advanced-stage HCC. Subsequently, we detected the mRNA and protein expression levels of RNF173 in 30 pairs of HCC and corresponding adjacent tissues, and the results showed that the expression of RNF173 was significantly decreased in tumor samples compared with adjacent tissues (Fig. 1C, D), which was consistent with the findings from the GEO database. Additionally, IHC staining and scoring of RNF173 were performed in 140 pairs of HCC and adjacent tissues, and we found that the protein expression level of RNF173 in HCC tumor tissues was significantly lower than in adjacent tissues (Fig. 1E, F). We then analyzed the correlation between RNF173 expression level and clinical pathological features of HCC patients. Kaplan–Meier survival analysis showed that patients with high expression of RNF173 (*n* = 48) had longer overall survival (OS) and recurrence-free survival (RFS) than those with low expression (*n* = 92) (Fig. 1G, H). Moreover, statistical analysis showed that low expression of RNF173 was significantly associated with adverse clinical pathological features, including tumor number (*p* = 0.03), tumor capsule (*p* = 0.017), tumor differentiation degree (*p* < 0.001), and Barcelona Clinic Liver Cancer (BCLC) stage (*p* = 0.022; Table 1). Cox regression analysis showed that tumor size, BCLC stage, and RNF173 expression were independent prognostic factors for OS, and tumor thrombus and RNF173 expression were independent prognostic factors for RFS (Supplementary Tables 1 and 2). These results suggest that RNF173 may play an important role in HCC and further investigation of its impact on invasion and metastasis of HCC is warranted.

**Table 1:** The association between RNF173 expression levels and various clinicopathological characteristics in patients with HCC

Variables		Number of patients		
		RNF173^low^	RNF173^high^	*p* value
Gender	Female	70	39	0.485
	Male	22	9	
Year	< 52	13	11	0.19
	≥ 52	79	37	
HBsAg	Negative	35	18	0.95
	Positive	57	30	
HCV	Negative	80	44	0.581
	Positive	12	4	
Tbil	< 22.2	75	35	0.239
	≥ 22.2	17	13	
ALB	< 30	2	0	0.546
	≥ 30	90	48	
ALT	< 40	55	32	0.425
	≥ 40	37	16	
AFP	< 20	49	25	0.895
	≥ 20	43	23	
Liver cirrhosis	No	12	7	0.801
	Yes	80	41	
Tumor number	Single	68	43	0.03 (*)
	Mutiple	24	5	
Embolus	Absence	58	36	0.153
	Present	34	12	
Tumor encapsulation	Absence	42	12	0.017 (*)
	Present	50	36	
Tumor differentiation	I + II	11	23	< 0.001 (***)
	III + IV	81	25	
Tumor size	< 5	44	25	0.632
	≥ 5	48	23	
BCLC stage	0 + A	53	37	0.022 (*)
	B + C	39	11	

### RNF173 modulates the invasiveness and metastasis of HCC cells

**Fig. 2:**
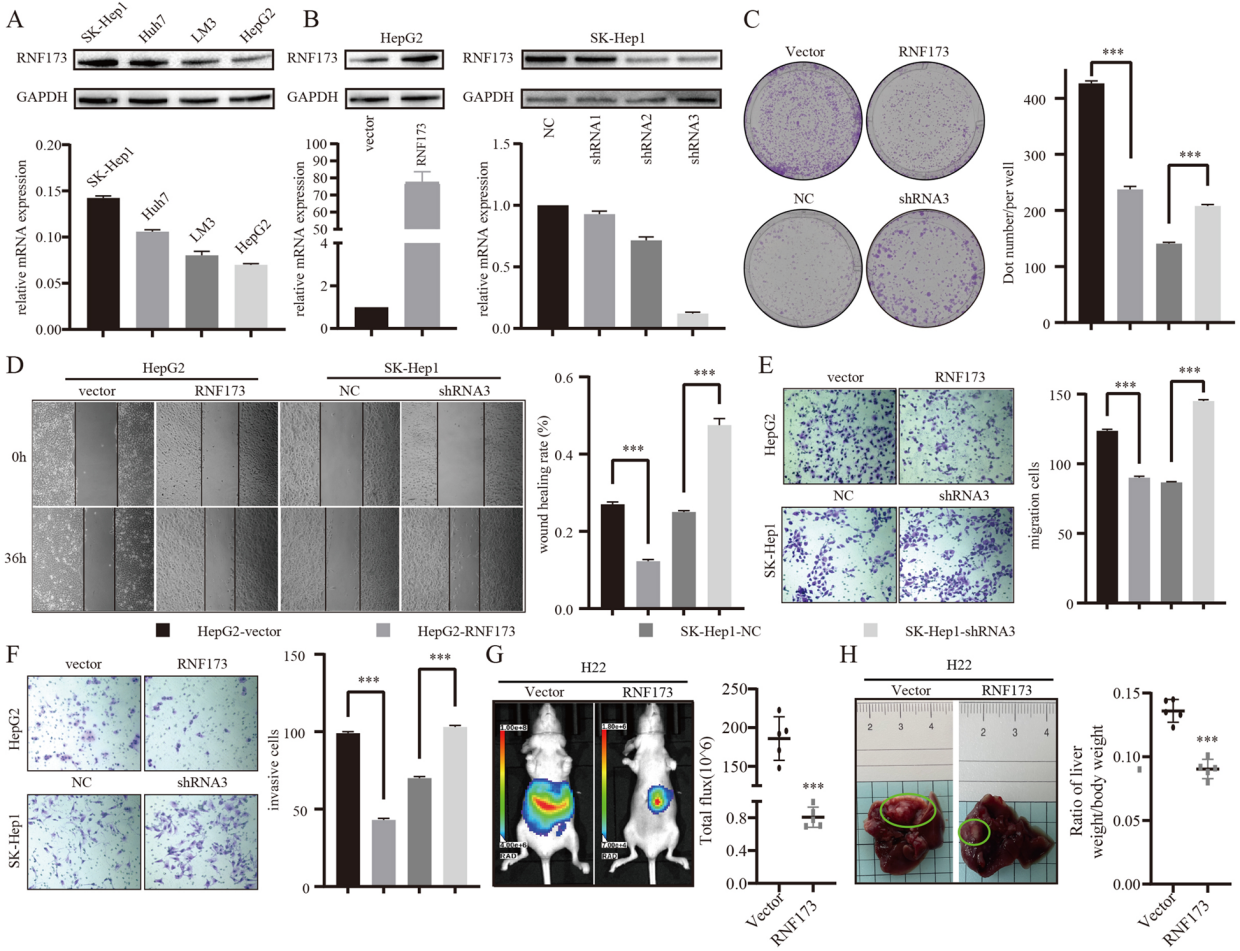
RNF173 regulates HCC proliferation, invasion, and migration in vitro and in vivo. (A) Expression levels of RNF173 in four HCC cell lines were evaluated by WB analysis. (B) Efficiency of RNF173 transfection in SK-Hep-1 and HepG2 cell lines was confirmed by WB and qRT-PCR. **C** Colony formation assay demonstrated that RNF173 knockdown enhanced cell proliferation, while RNF173 overexpression reduced it. **D**, **E** Wound healing and Transwell assays were used to evaluate the effect of RNF173 on cell migration. **F** Transwell assay was used to evaluate the effect of RNF173 on cell invasion. **G** Tumor growth in vivo was evaluated by measuring fluorescence signals in a live imaging system. (H) Liver weight to body weight ratio of mice in the orthotopic model. Scale bar: 100 μm. ***p < 0.001

The expression of RNF173 was evaluated in four HCC cell lines, HepG2, SK-Hep1, Huh7, and LM3, and we found that RNF173 was expressed at lower levels in HepG2 but at higher levels in SK-Hep1 cells (Fig. 2A). To investigate the role of RNF173 in the invasion and metastasis of liver cancer cells, we overexpressed RNF173 in HepG2 and knocked down RNF173 in SK-Hep1. The levels of RNF173 overexpression and knockdown were validated by WB and qRT-PCR (Fig. 2B). The colony formation experiment showed that the knockdown of RNF173 significantly enhanced the proliferation ability of HCC cells, while the overexpression of RNF173 significantly reduced it (Fig. 2C). The wound healing and Transwell assays revealed that RNF173 knockdown significantly enhanced the migration and invasion of HCC cells, while the overexpression of RNF173 reduced it (Fig. 2D). To further verify these results, we constructed an orthotopic tumor model in nude mice, and the results showed that the fluorescence signal of tumors detected in mice overexpressing RNF173 was significantly lower than that in the negative control group. The liver weight/body weight ratio in the RNF173 overexpression group was also significantly reduced. These in vitro and in vivo experiments suggest that RNF173 plays a crucial role in regulating the invasion and metastasis potential of HCC cells.

### RNF173 is involved in the process of epithelial-mesenchymal transition in HCC

**Fig. 3:**
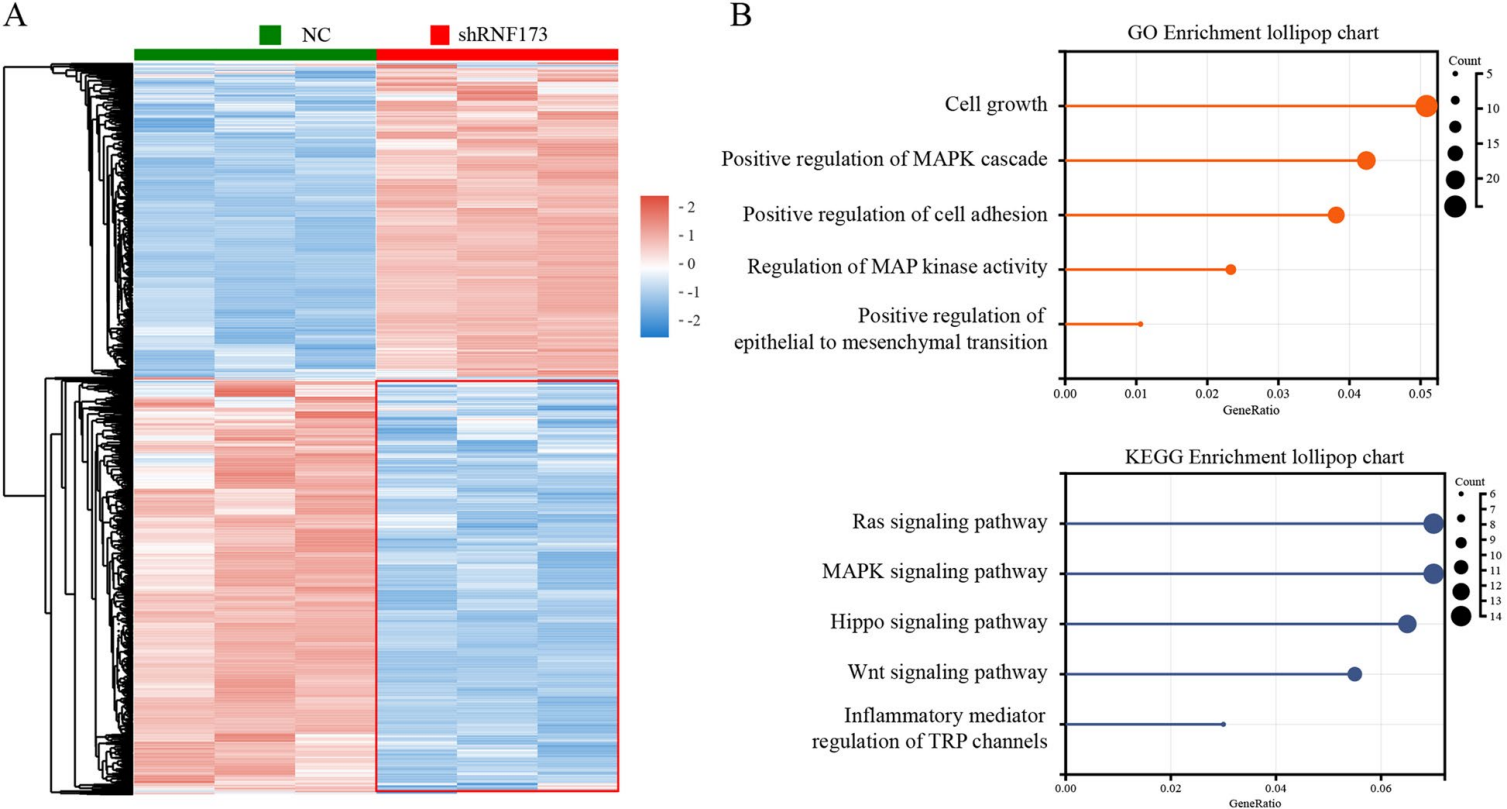
Transcriptomic analysis revealed that RNF173 participated in EMT. **A** RNA sequencing analysis of three samples each from the negative control and RNF173 knockdown groups resulted in a heatmap of 1055 differentially expressed genes (DEGs). (**B**) Functional enrichment analysis of 460 downregulated genes, including Gene Ontology (GO) and Kyoto Encyclopedia of Genes and Genomes (KEGG), revealed their involvement in EMT-related pathways

**Fig. 4:**
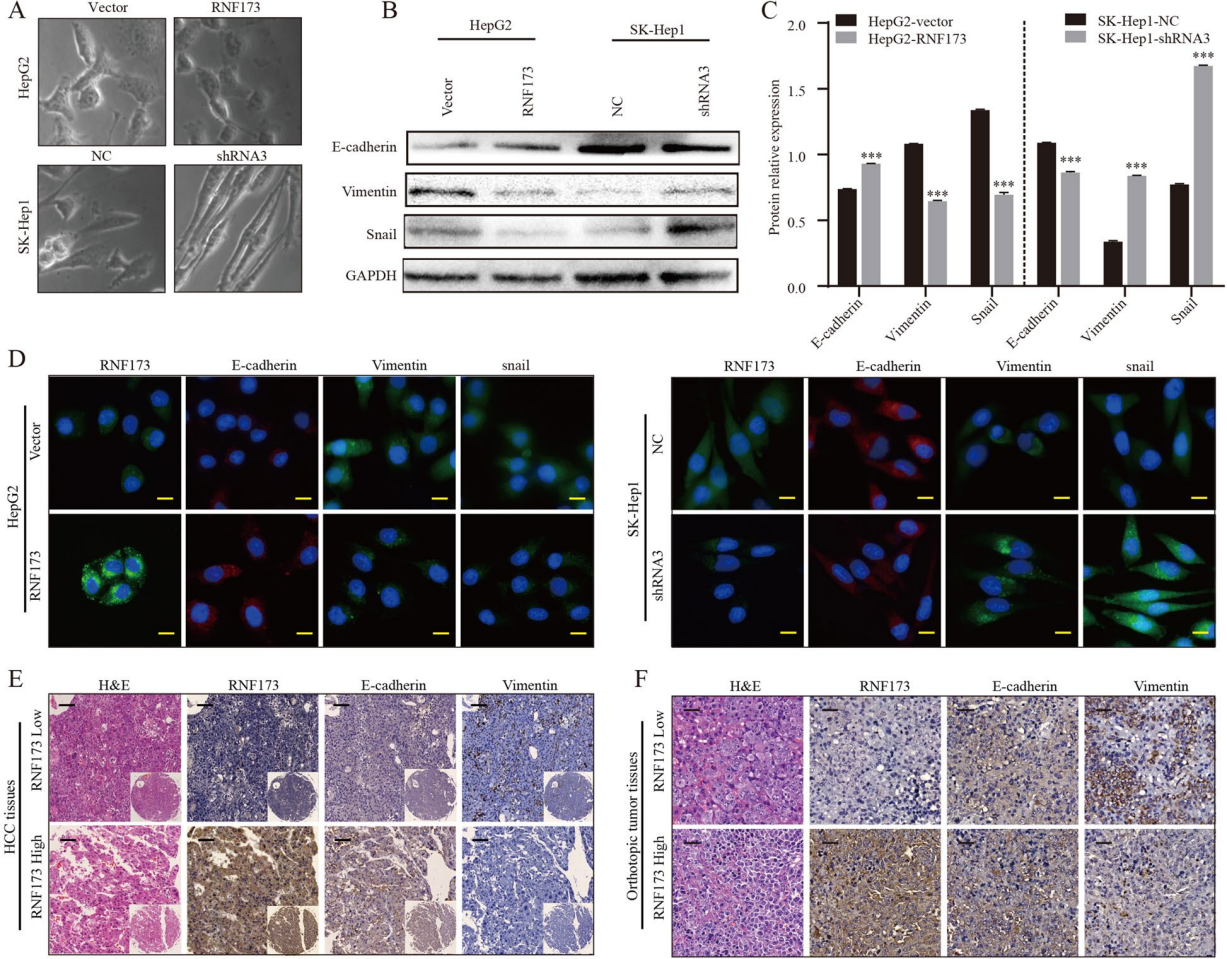
RNF173 was found to induce EMT in HCC cells. **A** The cellular morphology of four cell lines (SK-Hep1-NC, SK-Hep1-shRNA3, HepG2-vector, and HepG2-RNF173) was detected by a phase contrast microscope. **B** Expression of epithelial and mesenchymal markers was compared among SK-Hep1-NC, SK-Hep1-shRNA3, HepG2-vector, and HepG2-RNF173 cell lines. **C** Semi-quantitative analysis of western bolt by ImageJ. **D** Representative immunofluorescent images of RNF173, E-cadherin, Vimentin, and Snail in SK-Hep1-NC, SK-Hep1-shRNA3, HepG2-vector, and HepG2-RNF173. **E** Serial section and IHC staining showed expression of RNF173, E-cadherin, and Vimentin. **F** IHC staining from orthotopic tumor tissues showed expression of RNF173, E-cadherin, and Vimentin. Scale bars 50 μm. *** *p* < 0.001

To further explore the biological mechanisms, RNA-sequencing was performed to compare RNF173 knockdown cells to negative control cells. A total of 1055 genes were found to be significantly altered, with 460 downregulated and 565 upregulated genes between three RNF173-shRNA3 and three control samples, which were displayed in a heatmap (Fig. 3A). Based on these critical downregulated genes, the Gene Ontology (GO) enrichment of biological processes and the Kyoto Encyclopedia of Genes and Genomes (KEGG) enrichment were shown in a chart (Fig. 3B). The downregulated genes were mainly related to proliferation and metastasis pathways, including “epithelial to mesenchymal transition”, “Ras signaling pathway”, “MAPK signaling pathway”, and “Wnt signaling pathway”. These data showed that the downregulation of RNF173 might empower HCC cells to acquire mesenchymal phenotypes. EMT is a cellular process, characterized by increased migratory capacity and the absence of epithelial features [20]. To our surprise, we observed that downregulation of RNF173 make SK-Hep1 cells seemed more spindly (Fig. 4A). The expression of E-cadherin was found to be lower in SK-Hep1-shRNA3 cells compared to SK-Hep1-NC cells, while mesenchymal markers vimentin and snail were upregulated as RNF173 was knocked down (Fig. 4B, C). These results were confirmed by immunofluorescence (Fig. 4D) and IHC staining, which showed that HCC with lower levels of RNF173 tend to undergo EMT (Fig. 4E,F). These results indicate that RNF173 contributes to the malignant development of HCC by activating the EMT phenotype.

### RNF173 targets GRB2 as a ubiquitination substrate

**Fig. 5:**
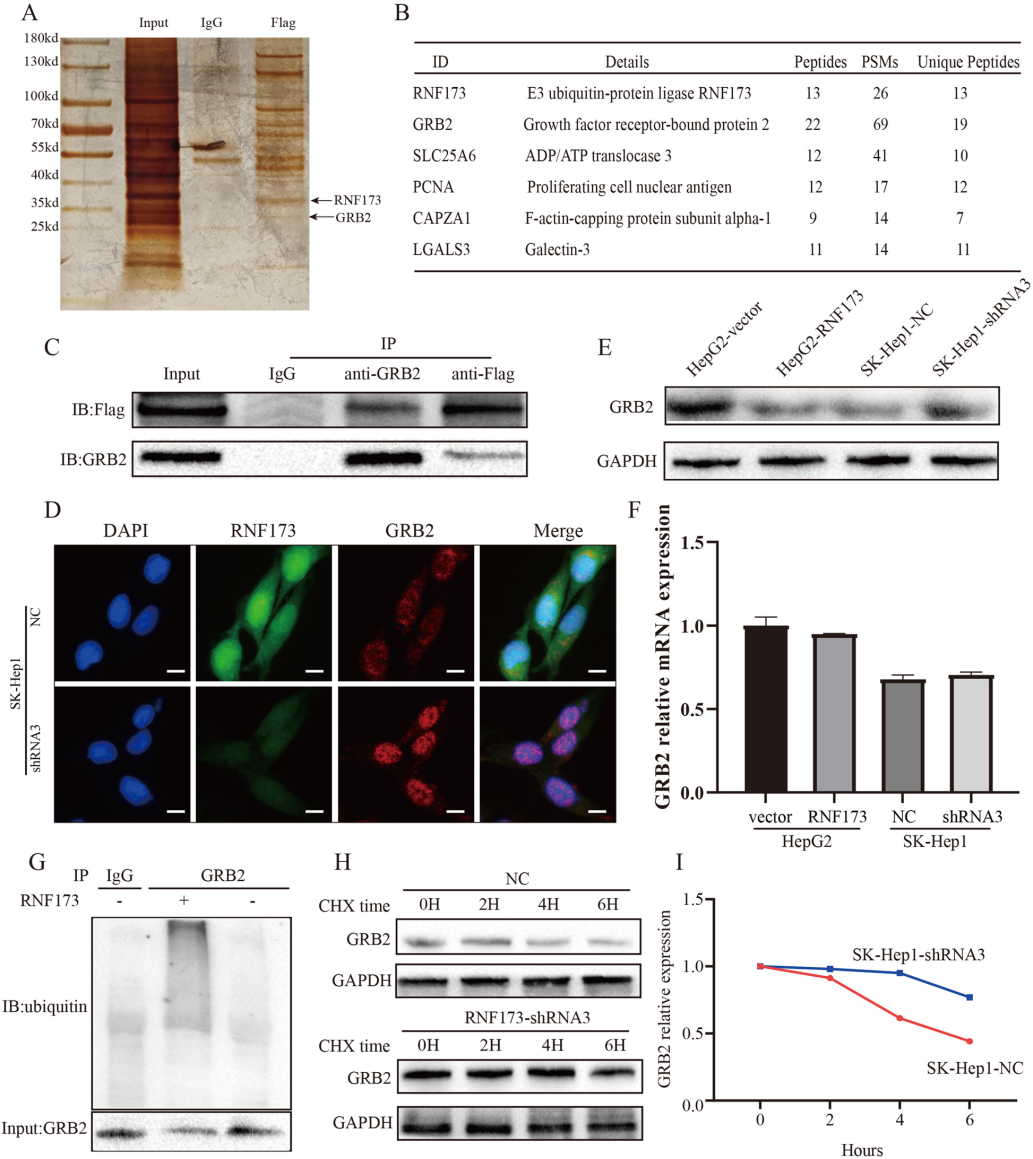
RNF173 interacted with GRB2 protein and promotes GRB2 protein degradation via ubiquitination. **A** Silver staining confirmed the presence of the RNF173 complex. **B** Mass spectrometry identified five potential interacting proteins. **C**, **D** Co-immunoprecipitation and immunofluorescence staining demonstrated the interaction between RNF173 and GRB2 protein. RNF173 (green), GRB2 (red), nucleus (blue). (E) WB showed that RNF173 affects the expression level of GRB2 protein. **F** qRT-PCR indicates that RNF173 did not affect the mRNA level of GRB2. **G** Immunoprecipitation of GRB2 from SK-Hep1-NC and SK-Hep1-shRNA3 cells, followed by immunoblotting with ubiquitin antibody, with the positive control being NC and the negative control being shRNA3. **H**, **I** The half-life of GRB2 was extended after RNF173 knockdown, as evidenced by cycloheximide treatment at 0, 2, 4, and 6 h. Scale bar, 50 μm

We conducted immunoprecipitation (IP) followed by silver staining (Fig. 5A) to investigate the molecular changes underlying the observed phenotype. The band patterns showed significant differences between the Flag and IgG groups. Through gel band mass spectrometry, we identified the top 5 potential targets, namely GRB2, SLC25A6, PCNA, CAPZA1, and LGALS3, after eliminating interfering factors (Fig. 5B). Among these targets, GRB2 is involved in the receptor tyrosine kinase signaling pathway, particularly the RAF/MEK/ERK pathway [21], which is consistent with the functional enrichment analysis mentioned earlier. Therefore, we hypothesized that there might be a regulatory relationship between RNF173 and GRB2. Subsequent co-immunoprecipitation and immunofluorescence staining experiments confirmed the formation of a complex between RNF173 and GRB2 (Fig. 5C, D). WB analysis showed that overexpression of RNF173 led to decreased expression of GRB2 protein (Fig. 5E), while RNF173 did not affect the mRNA level of GRB2 (Fig. 5F), indicating that post-transcriptional modification is an important factor in RNF173's regulation of GRB2. We obtained similar results in vivo experiments (Supplemental Fig. 1). Ubiquitination experiments suggested that the ubiquitination level of GRB2 was significantly decreased as RNF173 was knocked down (Fig. 5G). Treated with CHX, knockdown of RNF173 slowed down the degradation of GRB2 protein (Fig. 5H, I). These results demonstrate that RNF173 specifically binds to GRB2 and promotes its ubiquitination and degradation.

### RNF173 suppressed RAF/MEK/ERK pathway in HCC cells

**Fig. 6:**
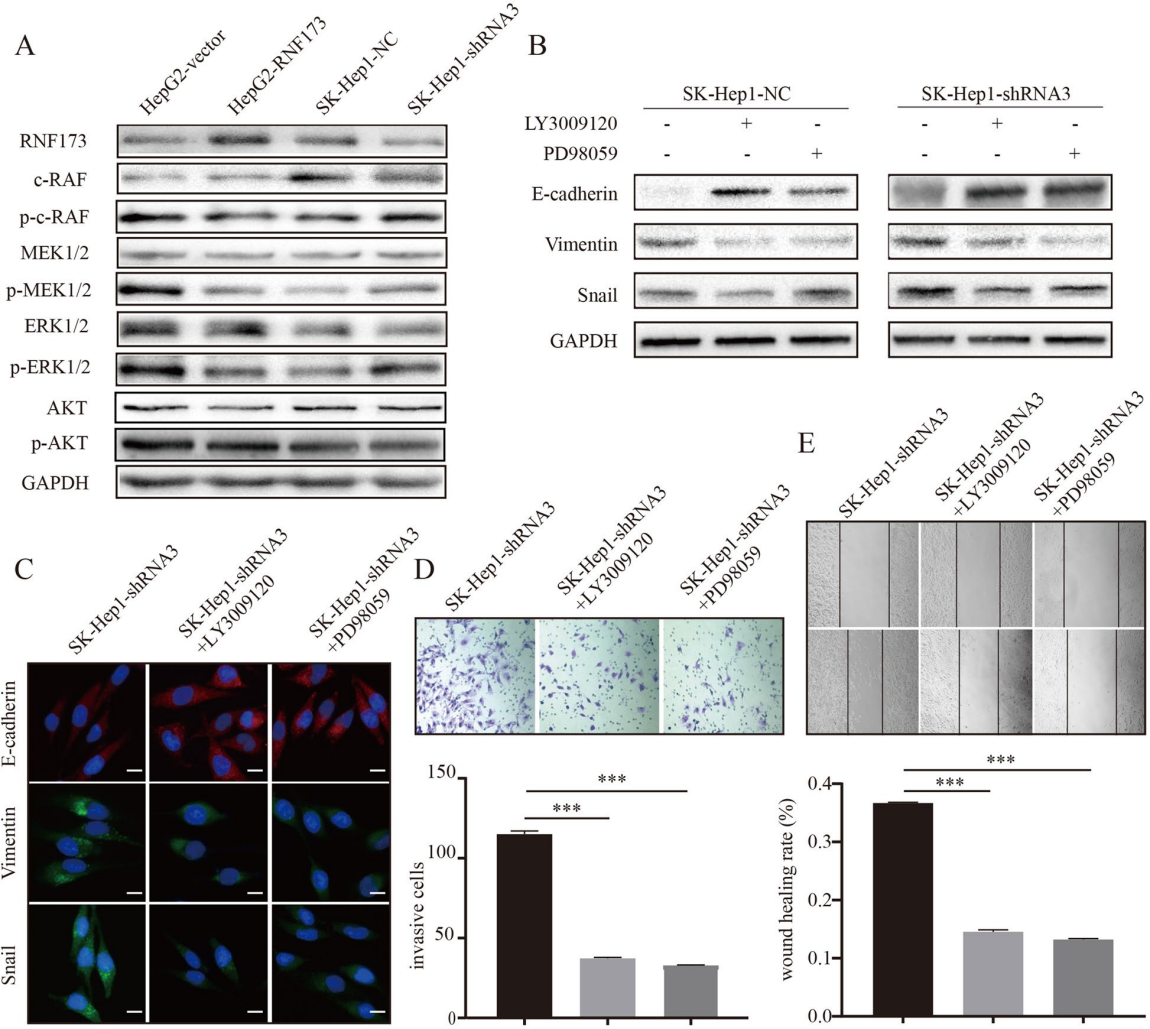
RNF173 knockdown promotes the RAF/MEK/ERK signaling pathway and HCC cell invasion and metastasis. **A** Western blot analysis of the effect of RNF173 knockdown on the phosphorylation levels of RAF, MEK1/2, and ERK1/2 in SK-Hep1-NC and SK-Hep1-shRNA3 cells. **B**, **C** Western blot and immunofluorescence analysis of the expression levels of epithelial marker E-cadherin and mesenchymal markers Vimentin and Snail in SK-Hep1-NC and SK-Hep2-shRNA3 cells after treatment with LY3009120 (10uM) and PD98059 (10uM) for 6 h. **D** Transwell assay showed that both LY3009120 and PD98059 reduced the invasion ability of SK-Hep1-shRNA3 cells. **E** Wound healing assay showed that both LY3009120 and PD98059 reduced the wound healing ability of SK-Hep1-shRNA3 cells. LY3009120, RAF inhibitor. PD98059, MEK inhibitor. ***p < 0.001

Based on the findings that RNF173 regulates the degradation of GRB2 and inhibits the RAF/MEK/ERK signaling pathway, we hypothesized that RNF173 may have a role in regulating the invasion and metastasis of HCC. To test this hypothesis, we analyzed the expression of key molecules in the pathway and found that knockdown of RNF173 increased the expression of phosphorylated c-RAF, phosphorylated MEK1/2, and phosphorylated ERK1/2 proteins, indicating activation of the RAF/MEK/ERK signaling pathway (Fig. 6A). In addition, treatment with small molecule inhibitors of RAF and MEK resulted in the upregulation of epithelial marker E-cadherin expression and downregulation of mesenchymal markers Vimentin and Snail expression (Fig. 6B, C). We further explored whether the regulation of the RAF/MEK/ERK pathway by RNF173 was involved in the modulation of HCC invasion and metastasis. Subsequently, we found that the small molecule inhibitors abrogated the promoting effect of RNF173 knockdown on the invasion and migration potential of HCC cells (Fig. 6D, E). These findings suggest that the regulation of the RAF/MEK/ERK signaling pathway by RNF173 via the ubiquitination and degradation of GRB2 plays a crucial role in the modulation of HCC proliferation, invasion and metastasis, as is showed in the graphical abstract.

### Clinical significance of RNF173 and GRB2 in patients with HCC

**Fig. 7:**
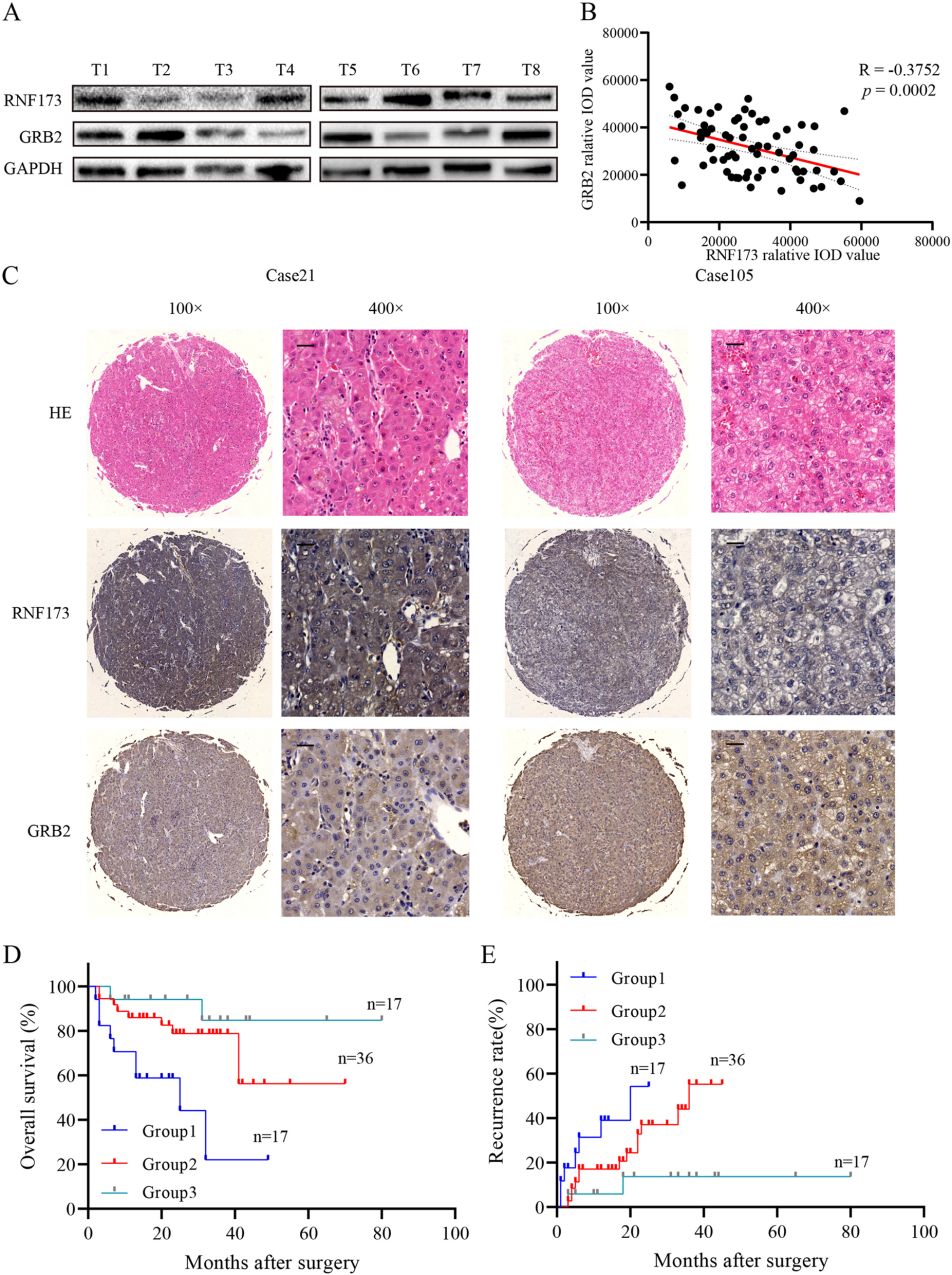
RNF173/GRB2 complex is an important factor in the prognosis of HCC patients. **A** Protein expression levels of RNF173 and GRB2 in HCC tumor tissues. **B** Correlation analysis of IHC staining intensity. **C** Representative HE and staining images of RNF173 and GRB2. **D**, **E** The overall survival rate and recurrence-free survival rate of HCC patients with high RNF173 and low GRB2 expression were the best. Patients were divided into three groups: Group 1 (*n* = 17) had low RNF173 and high GRB2 expression; Group 2 (*n* = 36) had both low or high expression of RNF173 and GRB2; Group 3 (*n* = 17) had high RNF173 and low GRB2 expression

Eventually, we investigated the expression relationship between RNF173 and GRB2 in HCC samples. We first examined the protein levels of RNF173 and GRB2 (Fig. 7A). Next, we randomly selected 70 samples and performed IHC staining to investigate the correlation between RNF173 and GRB2 expression levels. Our results revealed a significant negative correlation between RNF173 and GRB2 (Fig. 7B, C). Based on the positive expression levels of RNF173 and GRB2, we divided the patient cohort into three groups, where group 1 had low RNF173 and high GRB2, group 3 had high RNF173 and low GRB2, and group 2 had the remaining patients. We evaluated the effects of RNF173 and GRB2 on overall survival and recurrence rate and found significant differences among the three groups. Group 3 HCC patients had the best prognosis (Fig. 7D, E). In conclusion, our results suggest that co-expression of high RNF173 and low GRB2 may be a potential indicator for predicting overall survival and recurrence-free survival in HCC patients.

## Discussion

Although there have been advancements in the diagnosis and treatment of HCC, the prognosis for HCC patients remains poor [22, 23]. Previous studies have demonstrated the crucial regulatory role of E3 ubiquitin ligases in HCC. For instance, RNF128 enhances HCC proliferation, invasion, and migration by regulating the EGFR/ERK pathway [24]. Additionally, we found that overexpression of RNF38 can activate the TGF-β/SMAD3 signaling pathway, promoting HCC progression and epithelial-mesenchymal transition [11]. In this study, we investigated the differential expression of RNF173 in HCC compared to normal liver tissue and found that RNF173 expression was significantly and negatively correlated with HCC patient survival time and recurrence-free survival time. Furthermore, the expression level of RNF173 is an independent risk factor affecting HCC survival and recurrence. We employed lentiviral transfection technology to create stable cell lines with overexpression and knockdown of RNF173 in HepG2 and SK-Hep1 cells. Our in vitro and in vivo experiments confirmed that RNF173 inhibits HCC cell proliferation, invasion, and metastasis potential. We also revealed the underlying mechanism by which RNF173 inhibits HCC invasion and metastasis by ubiquitinating and degrading GRB2, suppressing the RAF/MEK/ERK pathway. Therefore, RNF173 plays a crucial regulatory role in HCC and may serve as a novel prognostic predictor and potential therapeutic target for liver cancer.

High invasiveness and metastasis of tumor cells are typically associated with the process of epithelial-mesenchymal transition [25, 26]. This transition refers to the loss of the gene expression pattern and cell morphology associated with epithelial cells and the acquisition of those required for invasiveness and metastasis. Microscopic examination of cells with knocked-out RNF173 revealed that they became more elongated. Further analysis showed that knocking out RNF173 resulted in upregulation of mesenchymal markers (Vimentin and Snail) and downregulation of epithelial markers (E-cadherin) both in vitro and in orthotopic tumor model, indicating that low expression of RNF173 can enhance HCC cell invasiveness and metastasis and induce EMT.

RNF173, a MARCH family protein [27], is known to regulate signal receptors. Previous research has indicated that RNF173 can ubiquitinate multiple interleukin receptor proteins and inhibit interleukin-related inflammatory responses [14–17]. However, the role of RNF173 in liver cancer regulation has not been clearly understood. Here, we used mass spectrometry and ubiquitination experiments to identify that RNF173 participates in the ubiquitination and degradation of GRB2. Our findings suggest that RNF173 ubiquitinates GRB2 without altering its transcriptional level, and knocking down RNF173 impaired the degradation of GRB2 and extends its half-life. Our correlation analysis in HCC tumor tissues revealed a significant negative correlation between the protein expression levels of RNF173 and GRB2. Clinically, we observed that HCC patients with high RNF173 expression and low GRB2 expression had the best prognosis. Previous studies have identified GRB2 as an adapter protein that links surface receptors to downstream targets [28–31]. GRB2 is known to promote the migration of non-small cell lung cancer (NSCLC) cells and has been identified as a potential target for NSCLC therapy [32]. In HCC tissues, GRB2 is significantly upregulated and its expression is negatively correlated with overall survival in HCC patients [33]. Besides, GRB2 plays a critical role in linking growth factor RTK to the RAF/MEK/ERK signaling pathway [34–36] by recruiting and activating nucleotide exchange factors for guanine to activate the RAS signaling pathway, leading to phosphorylation of serine/threonine kinases (RAF) and downstream molecules [21]. Inhibition of the GRB2/MAPK pathway has been shown to induce cell senescence [37, 38], while overexpression of GRB2 contributes to epithelial-mesenchymal transition and promotes cell migration in A549 cells [39]. In this study, RNA sequencing was performed on the RNF173 knockdown group and the control group, and functional enrichment of differentially expressed genes indicated that RNF173 may regulate the RAF/MEK/ERK signaling pathway. Subsequent experiments confirmed that RNF173 ubiquitinates and degrades GRB2, which regulates the RAF/MEK/ERK pathway, thereby inhibiting HCC cell proliferation, invasion, and migration. This study still has some limitations. Firstly, this study did not investigate the specific molecular binding domains between RNF173 and GRB2. Secondly, although meaningful results were obtained in cellular and animal models, further research is required before applying these findings to clinical translation.

## Conclusion

In conclusion, this study provides insights into the clinical significance of RNF173 expression in HCC and uncovers its mechanism of action in suppressing HCC proliferation, invasion, and migration by regulating the RAF/MEK/ERK signaling pathway through ubiquitination-mediated degradation of GRB2. The downregulation of RNF173 in HCC is associated with an EMT phenotype and poor prognosis. Therefore, RNF173 may serve as a promising diagnostic biomarker and prognostic predictor for predicting the survival rate of HCC patients, guiding patient follow-up, and informing individualized treatment plans. Moreover, targeting RNF173 and its downstream signaling pathways may represent a potential therapeutic strategy for the treatment of HCC. Further research is needed to fully understand how RNF173 may be affecting immune cells and their interactions with cancer cells in in tumor immune microenvironment of HCC.

### Supplementary Information

**Additional file 1: Supplemental Figure 1.** Immunohistochemical staining revealed the expression of RNF173 and GRB2 in the tumor tissues of mice in both the vector and RNF173 overexpression groups. **Table S1&S2.** Univariate and multivariate analysis of OS and RFS in HCC. **Table S3.** The RNF173 shRNA sequence in this study. **Table S4.** The primers used in this study. **Table S5.** A list of antibodies used in this study.

## Data Availability

All data underlying the study are available from the corresponding authors on. reasonable request.

## Abbreviations

HCC: Hepatocellular carcinoma
RNF173: RING Finger Protein 173
GRB2: Growth Factor Receptor Bound Protein 2
EMT: Epithelial-mesenchymal transition
RAF: Proto-Oncogene Serine/Threonine Protein Kinase
MEK: Mitogen-Activated Protein Kinase Kinase
ERK: Mitogen-Activated Protein Kinase
AKT: Serine/Threonine Kinase
OS: Overall survival
RFS: Recurrence-free survival
shRNA: Short hairpin RNA
IHC: Immunohistochemistry
Co‐IP: Coimmunoprecipitation
WB: Western blotting
