# Supplementary material for: RNF173 suppresses RAF/MEK/ERK signaling to regulate invasion and metastasis via GRB2 ubiquitination in Hepatocellular Carcinoma
Source: Cell Commun Signal. 2023 Aug 25;21:224. doi: 10.1186/s12964-023-01241-x (PMC10464048; doi:10.1186/s12964-023-01241-x)
Supplement: Supplementary file 2 — Additional file 1: Supplemental Figure 1. Immunohistochemical staining revealed the expression of RNF173 and GRB2 in the tumor tissues of mice in both the vector and RNF173 overexpression groups. Table S1&S2. Univariate and multivariate analysis of OS and RFS in HCC. Table S3. The RNF173 shRNA sequence in this study. Table S4. The primers used in this study. Table S5. A list of antibodies used in this study. [file 12964_2023_1241_MOESM1_ESM.docx]

**Supplemental Figure 1 Immunohistochemical staining revealed the expression of RNF173 and GRB2 in the tumor tissues of mice in both the vector and RNF173 overexpression groups.**

**
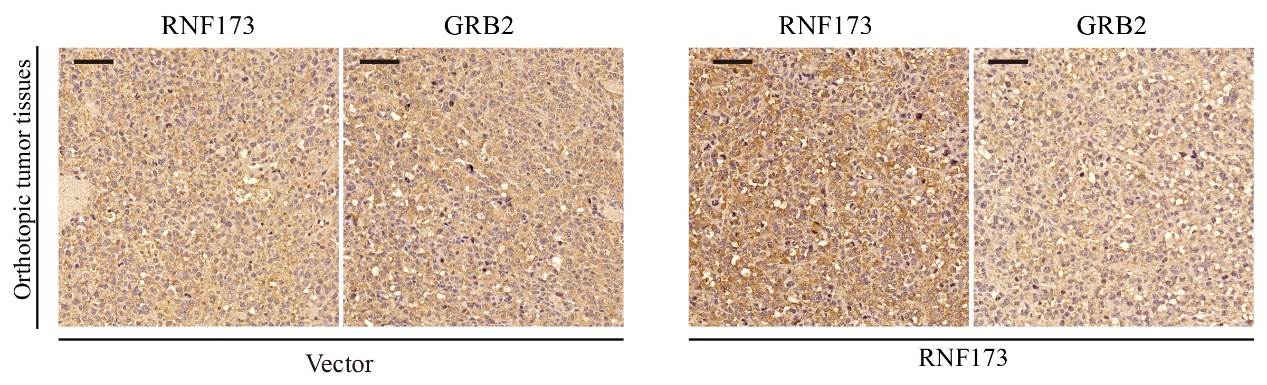
**

**Supplementary table**

**Table S1** Univariate and multivariate analysis of OS in HCC


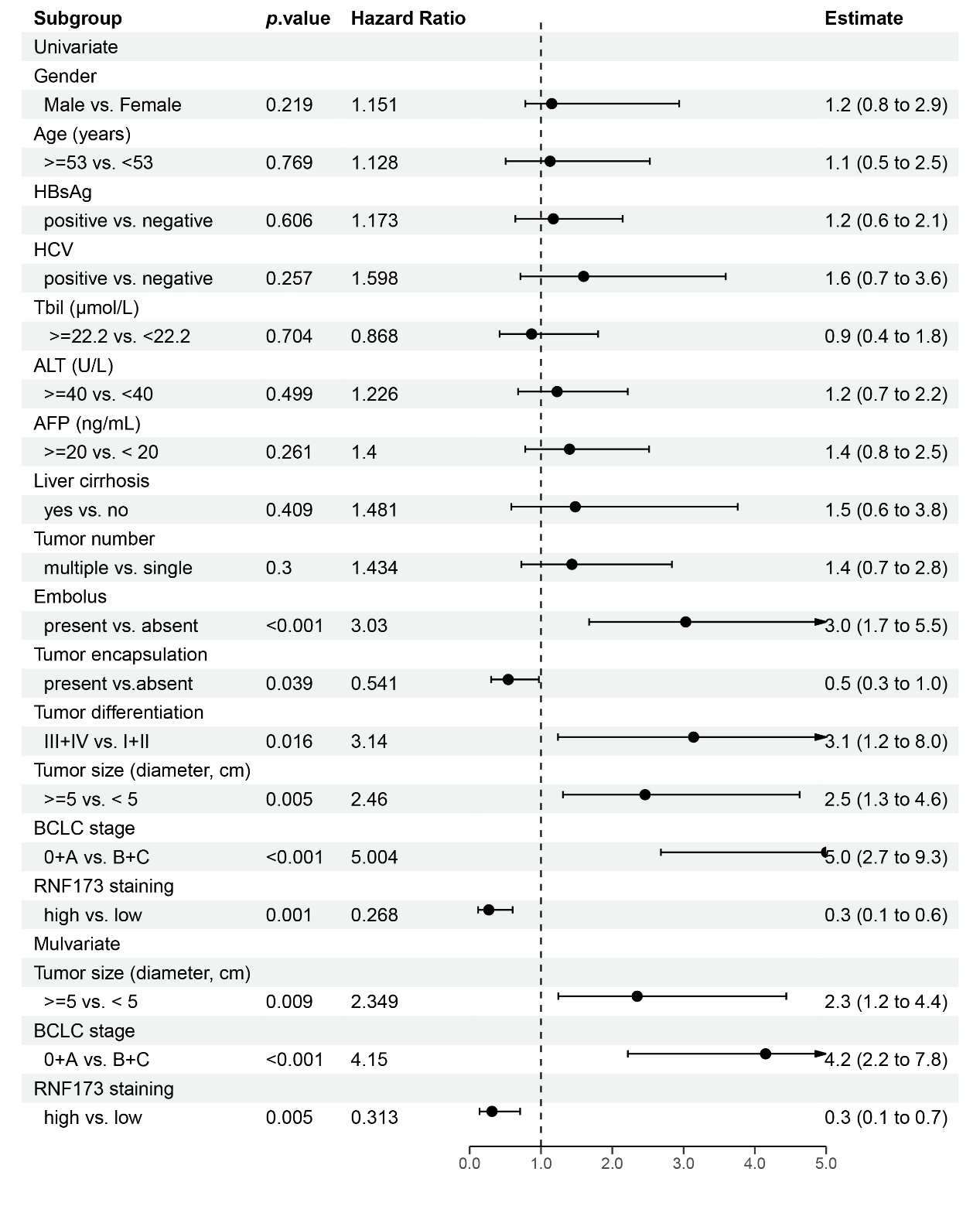


**Table S2** Univariate and multivariate analysis of RFS in HCC


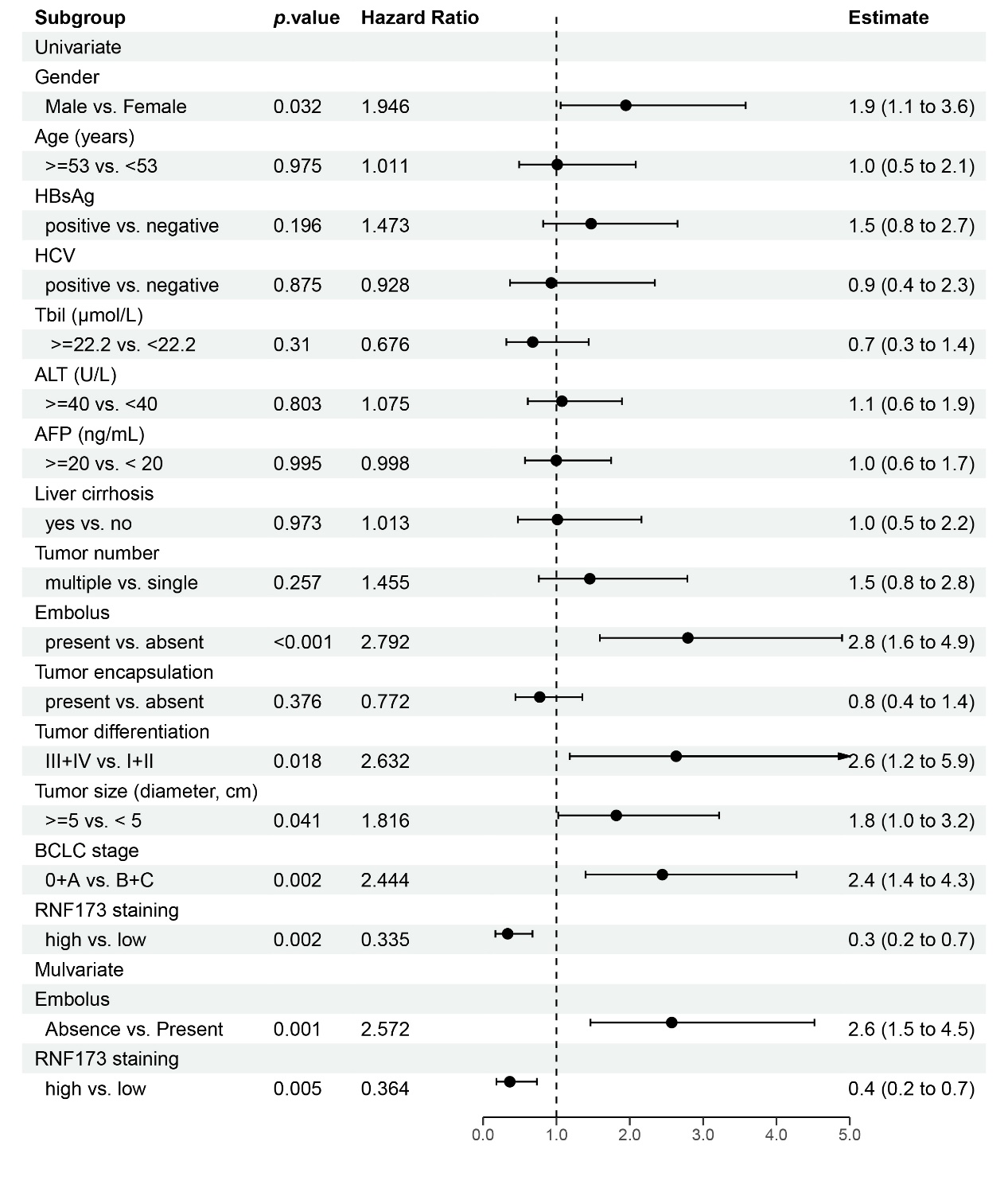


**Table S3** The RNF173 shRNA sequence in this study

| Gene | No. | Target sequences |
| --- | --- | --- |
| RNF173 | NC  shRNA1 | TTCTCCGAACGTGTCACGT  GGTGAAGACGGTGGAGGATTG |
|  | shRNA2 | GCCGCAGTATGTCATGCAAGT |
|  | shRNA3 | GGTTTGCAGTCGAGCGCAAAC |

**Table S4** The primers used in this study

| Primers name |  | Sequence (5’-3’) |
| --- | --- | --- |
| RNF173 | Forward | TGGCGACATGGTGTGCTTCTTG |
|  | Reverse | AATAGTGAAGAGTGCGACAGTGAGTG |
| GRB2 | Forward | TCCTCTGGGTGGTGAAGTTCAATTC |
|  | Reverse | GCTGTGGCACCTGTTCTATGTCC |
| GAPDH | Forward | GTCTCCTCTGACTTCAACAGCG |
|  | Reverse | ACCACCCTGTTGCTGTAGCCAA |

**Table S5** A list of antibodies used in this study

| Antibodies | Company | Cat.No | Mol weight (kDa) | Dilution |
| --- | --- | --- | --- | --- |
| RNF173 | Abcam | ab137261 | 30 | 1:500(WB)  1:50(IHC) |
| RNF173 | NOVUS | NBP1-81260 | - | 1:400(IF) |
| GRB2 | Proteintech | 10254-2-AP | 28 | 1:1000(WB)  1:100(IHC) |
| GRB2 | SANTA CRUZ | sc-8034 | - | 1:400(IF) |
| E-cadherin | CST | 14472 | 120 | 1:1000(WB)  1:100(IHC) |
| E-cadherin | Proteintech | 20874-1-AP | - | 1:600(IF) |
| Vimentin | CST | 5741 | 60 | 1:1000(WB)  1:200(IHC)  1:200(IF) |
| Snail | CST | 13099-1-AP | 29 | 1:1000(WB) |
| Snail | Sino | 101167-T10 | - | 1:500(IF) |
| c-Raf | CST | 53745 | 74 | 1:1000(WB) |
| Phospho-c-Raf | CST | 20011 | 74 | 1:1000(WB) |
| MEK1/2 | CST | 4694 | 45 | 1:1000(WB) |
| Phospho-MEK1/2 | CST | 9154 | 45 | 1:1000(WB) |
| Erk1/2 | CST | 4695 | 42 | 1:1000(WB) |
| Phospho- Erk1/2 | CST | 4370 | 42 | 1:1000(WB) |
| Akt | CST | 9272 | 60 | 1:1000(WB) |
| Phospho-Akt | CST | 4060 | 60 | 1:1000(WB) |
| Ubiquitin | Proteintech | 10201-2-AP | - | 1:1000(WB) |
| Alexa Fluor 488 AffiniPure Donkey anti-Rabbit IgG (H+L) | Yeasen | 34212ES60 | - | 1:200(IF) |
| Alexa Fluor 555 AffiniPure Donkey anti-Mouse-IgG (H+L) | Beyotime | A0460 | - | 1:200(IF) |
| GAPDH | Proteintech | 60004-1-Ig | 36 | 1:100000(WB) |
| Anti-rabbit IgG- | CST | 7074 | - | 1:2000(WB) |
| Anti-mouse IgG | CST | 7076 | - | 1:2000(WB) |
